# A multivariate hierarchical Bayesian approach to measuring agreement in repeated measurement method comparison studies

**DOI:** 10.1186/1471-2288-9-6

**Published:** 2009-01-22

**Authors:** Philip J Schluter

**Affiliations:** 1 Monash University, Accident Research Centre, Clayton, Melbourne, Victoria 3800, Australia; 2 AUT University, School of Public Health and Psychosocial Studies, Private Bag 92006, Auckland 1142, New Zealand; 3 The University of Queensland, School of Nursing and Midwifery, Brisbane, Queensland 4072, Australia

## Abstract

**Background:**

Assessing agreement in method comparison studies depends on two fundamentally important components; validity (the between method agreement) and reproducibility (the within method agreement). The Bland-Altman limits of agreement technique is one of the favoured approaches in medical literature for assessing between method validity. However, few researchers have adopted this approach for the assessment of both validity and reproducibility. This may be partly due to a lack of a flexible, easily implemented and readily available statistical machinery to analyse repeated measurement method comparison data.

**Methods:**

Adopting the Bland-Altman framework, but using Bayesian methods, we present this statistical machinery. Two multivariate hierarchical Bayesian models are advocated, one which assumes that the underlying values for subjects remain static (exchangeable replicates) and one which assumes that the underlying values can change between repeated measurements (non-exchangeable replicates).

**Results:**

We illustrate the salient advantages of these models using two separate datasets that have been previously analysed and presented; (i) assuming static underlying values analysed using both multivariate hierarchical Bayesian models, and (ii) assuming each subject's underlying value is continually changing quantity and analysed using the non-exchangeable replicate multivariate hierarchical Bayesian model.

**Conclusion:**

These easily implemented models allow for full parameter uncertainty, simultaneous method comparison, handle unbalanced or missing data, and provide estimates and credible regions for all the parameters of interest. Computer code for the analyses in also presented, provided in the freely available and currently cost free software package WinBUGS.

## Background

Accurate measurement of the variable of interest is fundamentally important in any health research or practice setting. However, it is widely recognised that measurements simultaneously made on the same subject or specimen by different instruments, methods or observers invariably yield different empirical values. As such, evaluation of measurement quality is a central issue in deciding the utility of any instrument, method or observer [1]. Measurement validity and reproducibility are essential elements in determining this quality. Validity is the degree to which a measurement measures what it purports to measure and reproducibility is the degree to which a measurement provides the same result each time it is performed on a given subject or specimen [2]. Reproducibility is invariably assessed using agreement analysis of within (intra) and between (inter) instrument, method or observer measurement comparison studies. For ease of exposition, we shall refer to instrument, method or observer comparisons simply as method comparisons hereafter.

In measurement method comparison studies, the main interest is to determine whether the measurements made on the same subject or specimen by different methods can be used interchangeably [3,4]. Typically, measurement method comparison studies are motivated when newer, less invasive, safer or cheaper measurement techniques become available and we wish to assess the agreement between them and some "gold standard" or existing technique. Lack of agreement between different methods is inevitable, as all instruments measure with some error, but the questions of interest is by how much do the methods disagree and is this difference important? Multiple statistical strategies exist that can be used to assess this form of agreement [3], including the Bland-Altman limits of agreement approach [4-6], regression techniques [7,8], nonparametric methods [6], and survival-agreement plots [9]. As the Bland-Altman limits of agreement approach is simple to employ, practical, and detects bias, it has become the preferred method within health research in recent years [3,10].

In its simplest form, the Bland-Altman limits of agreement approach compares unreplicated paired measurements between two methods over a number of subjects or specimens [5]. A graphical depiction of differences between paired observations versus their average is typically presented in a scatter-plot. Generally, superimposed on the scatter-plot is a horizontal line indicating bias (calculated as the mean difference between measurement pairs, $\bar{d}$) and horizontal lines giving the 95% limits of agreement (calculated, assuming the differences are approximately normally distributed, using the standard deviation of the differences, *s*, via $\bar{d}$ ± 1.96 × *s*). The limits of agreement define the range within which 95% of the differences between measurements by the two methods are predicted to lie. The scatter-plot is used to determine whether any patterns exist in the data, thereby potentially violating the method's assumptions, or revealing whether data transformation is necessary. A histogram of the paired differences ordinarily accompanies the scatter-plot and should be normally distributed. Only once these checks are completed and assumptions satisfied can an assessment be made to the acceptability of the quantified level of agreement for clinical or epidemiological purposes.

At times, however, more than two measurement observers or instruments are of particular interest and simultaneously assessed. For example, the research question that motivated this paper was where should pedometers (devices for counting steps) be positioned on children (left hip, right hip, or the back) to give best agreement with observed step counts (the 'gold-standard')? Most statistical approaches use separate pair-wise comparisons of methods in these situations [6]. However, this situation lends itself to a multivariate form of analysis.

Measurement repeatability is important in measurement method comparison studies because it limits the amount of agreement which is possible [5,8]. If methods have poor repeatability then there is likely to be considerable variation in repeated measurements on the same subject or specimen thus resulting in poor agreement. Given this importance of repeatability, Bland and Altman advocated in their 1986 paper a design that allowed estimation of both limits of agreement between two methods and coefficients of repeatability for each method [5]. However, in 2003, these authors note, to their chagrin, that this approach has not been widely adopted by researchers [4].

It might be opined that one of the primary reasons why so few repeatable measurement studies have been undertaken is due to the lack of readily available and easily implemented statistical machinery for the analysis of such data, especially if the number of replicates is unbalanced or some data are missing. In an effort to circumvent this problem, Bland and Altman in 1999 presented analytical techniques similar to their limits of agreement approach to quantify the repeatability of a method where the underlying values for subjects remain static over replications (where values can be considered as being exchangeable) using one-way analysis of variance methods and variance component techniques [6]. They also described a method for analysing replicated data in pairs where several pairs of measurements are made by two methods on each subject or specimen where the underlying true value changes from pair to pair (here the measurement pairs are consider non-exchangeable). While most of these methods are straightforward and relatively easily implemented, some of the assumptions are restrictive and potentially unrealistic [3,8]. Moreover, should there been more than two methods under consideration then the proposed techniques are not easily generalised to simultaneously assess these methods.

In 2004, Carstensen described more general regression and variance component methods for the analysis of such data [8]. While conceptually appealing, these methods can be difficult to implement thereby limiting their utility. Recent energies by Carstensen and colleagues have been to report simplified versions of his methods and develop new techniques with greater practical utility [11].

Until now, there have been no published Bayesian methods focusing on measurement method comparison studies. This is perhaps surprising given the increased utilisation of Bayesian techniques and their apparent suitability to this type of problem. In a complementary analysis of repeated measurements of paired outcomes data, a multivariate hierarchical Bayesian method has already been successfully employed and many salient advantages described [12]. Bayesian methods have the advantage of embodying and yielding parameter distributions rather than using point-estimates; the balance of the data is unimportant, multiple methods can be compared simultaneously in a single analysis, they are readily implemented and interpreted; and, they are easily generalised to more complex study designs and hierarchies [12-14]. As bounded prior distributions can be incorporated into Bayesian analyses, sensible posterior distributions and credible regions can be derived for all parameters of interest, and many convergence or computational problems associated with non-Bayesian methods can be eliminated. Moreover, the methods are easily extended to include informative prior distributions, allow covariates and subject subgroup structures to be incorporated, and provide probabilistic subject specific and overall group results [12-14].

Based on the limits of agreement approach framework, this paper advocates assessing agreement in repeated measurement method comparison studies using a fully parametric multivariate hierarchical Bayesian approach. Two models are proposed in this paper; the selection of the appropriate analysis depends on the underlying values of the variable of interest. Like that propounded by Bland and Altman in 1999, one model assumes exchangeable values for each subject while the other accommodates non-exchangeable values [6]. Section 2 describes the two related statistical models we employ. Using data previously presented and analysed by Bland and Altman [6] and new data from Oliver and colleagues [15], we illustrate the use of the proposed models with numerical results in Section 3. Concluding remarks are then presented in Section 4.

## Methods

### Specification of the hierarchical Bayesian models

Depending on the underlying values of the variable of interest, two models are considered, namely: (i) an exchangeable multivariate hierarchical Bayesian model (*HB*_1_); and (ii) a non-exchangeable multivariate hierarchical Bayesian model (*HB*_2_).

### Exchangeable multivariate hierarchical Bayesian model (*HB*_1_)

Consider a measurement method comparison study that is conducted using *m *= 1,..., *M *methods, *M *≥ 2 on *i *= 1,..., *N *subjects and that for each method and subject *r *= 1,..., *R*_*mi *_repeated measurements are made. Note that the number of repeated measurements can vary by method and subject, and measurements for the *M *methods need not be made simultaneously as the underlying values for subjects are assumed to remain static over all replications. Let *x*_*mir *_denote the observed value obtained using method *m *on subject *i *for replicate *r*. Suppose that the repeated values on each subject within each method can be considered exchangeably (i.e. the order of $x_{mi1},\ldots ,x_{miR_{mi}}$ values for any given method *m *and subject *i *are interchangeable) then an intuitive approach is to model the within and between subject levels using a hierarchical model.

Exploiting the exchangeability assumption, we assume that the first or observation level of the model can by represented by

*x*_*mir *_~
*MVN*(*μ*_*mi*_,
**Θ**)

where *MVN*(.,.) denotes a multivariate normal distribution, *μ*_*mi *_is the underlying mean value for method *m *and subject *i*, and **Θ **is the *M *× *M *dimensional covariance matrix made at this observation level. Further, we assume that the second or subject level of the hierarchical model can given by

*μ*_*mi *_~
*MVN*(*θ*_*m*_,
**Ω**)

where *θ*_*m *_is the overall population means for method *m*, and **Ω **is the *M *× *M *dimensional covariance matrix at the subject level. To complete the full parameterization, prior distributions need to be specified for *θ*_1_,..., *θ*_*M*_, **Θ **and **Ω **and will depend on the information available.

It is straightforward to see that the bias between any two methods, *y *and *z*, such that *y *= 1,..., *M*, *z *= 1,..., *M *and *y *≠ *z*, is given by

*B*_(*y*, *z*) _=
*θ*_*y *_-
*θ*_*z*_.

Note that *B*_(*y*, *z*) _= -*B*_(*z*, *y*) _and so it is convenient to limit *y *and *z*, such that *y *= 1,..., *M *- 1, *z *= 2,..., *M *and *y *<*z *This formulation of *B*_(*y*, *z*) _implies that the distribution of bias remains constant over the full measurement range for methods *y *and *z*. If this assumption is found to be too restrictive, then it may be relaxed provided sufficient information is available. We note that the subjects chosen within the sample may not necessarily themselves follow a normal distribution as measurement method comparison studies often select subjects that give a wide distribution of the quantity measured rather than some random selection. However, the population of subjects that the selected subjects are drawn from can frequently be assumed to follow a normal distribution and so this assumption is often reasonable; although, with evidence to the contrary, other distributional forms may also be adopted.

The specification of the *M *× *M *dimensional covariance matrix **Θ **at the first level forces the within subject variance of measurements for method *m *(denoted by $s_{within\left(m\right)}^{2}$) and the covariance of measurements between two methods *y *and *z *(denoted by $\tau_{\left(y,z\right)}^{2}$) to be identically distributed across all subjects *i *= 1,..., *N*. If there is good reason to suspect that these assumptions are too restrictive or flexible, then they can be modified or difference covariance specifications formulated. For instance, with exchangeable measurements made simultaneously across methods then it makes intuitive sense for the off-diagonal elements of the covariance matrix **Θ **to be unrestricted. However, should the measurements for the *M *methods be made at different times, independently from each other, then the off-diagonal elements of **Θ **might be constrained to 0. The *M *× *M *dimensional covariance matrix **Ω **at the second level gives the between subject variance (denoted by $s_{between\left(m\right)}^{2}$) and covariance between methods.

Through simulation, this specification of the hierarchical Bayesian model allows marginal distributions for a number of parameters of interest to be easily determined, thereby providing means for estimation of means and credible regions. In particular, for model *m *the $s_{within\left(m\right)}^{2}$ = **Θ**_*mm*_, $s_{between\left(m\right)}^{2}$ = **Ω**_*mm *_and the intra-class correlation coefficient *ICC*_*m *_= **Ω**_*mm*_/(**Ω**_*mm *_+ **Θ**_*mm*_) are readily obtained. Although widely used, we note that the utility of intra-class correlation coefficients in method comparison studies has been questioned [10]. To compare against non-Bayesian approaches, it is also of interest to report the within subject covariance of measurements between methods *y *and *z*, $\tau_{\left(y,z\right)}^{2}$ = **Θ**_*yz*_.

### Non-exchangeable multivariate hierarchical Bayesian model (*HB*_2_)

When we have repeated measurements by *M *methods made simultaneously on the same subject where the subject's underlying value could be a continually changing quantity, we can estimate the limits of agreement by modelling measurement pair differences [6].

As before, we let *x*_*mir *_denote the observed value obtained using method *m *(*m *= 1,..., *M*) on subject *i *(*i *= 1,..., *N*) for replicate *r *(*r *= 1,..., *R*_*mi*_) Now let

*d*_(*y*, *z*)*ir *_=
*x*_*yir *_-
*x*_*zir*_

be the difference between observed values of two methods, *y *and *z*, such that *y *= 1,..., *M *- 1, *z *= 2,..., *M *and *y *<*z*, for subject *i *and repeated measure *r*. Note, here, that each wave of repeated measurements are made simultaneously by all *M *methods under investigation. Thus the number of repeated measurements can vary by subject but not by method within subject, and *d*_(*y*, *z*)*ir *_will be missing if either or both *x*_*yir *_and *x*_*zir *_are missing.

For *M *different methods we have *M*!/2!(*M *- 2)! different pair-wise comparisons. However, if there is no or negligible missing data for any of the *M *different methods, then there are linear dependences in these differences over all potential different pair-wise comparisons and only *M *- 1 comparisons are needed to determine the rest. For example, if we have three methods J, R and S, then we have 3!/2!(3 - 2)! = 3 different pair-wise comparisons, namely: J & R, J & S, and R & S. However the pair-wise comparison of R & S, for example, is dependent on pair-wise comparisons J & R and J & S, and so need not be explicitly modelled. Indeed, if all three pair-wise comparisons were to be simultaneously numerically simulated then it might be expected that convergence would be poor and autocorrelation high in the relevant parameters. If there is non-negligible missing data for any of the methods, then care must be given to determining which pair-wise comparisons should be modelled and their associated dependences considered. In practice, if one method systematically provided non-negligible missing data relative to other methods, then this would probably be grounds enough to question the utility of this method.

Let us assume that there is no missing data for each of the *M *methods and we model the pair-wise differences between method *y *= 1 and methods *z *= 2,..., *M*. All other pair-wise comparisons we might consider can be derived from these *M *- 1 comparisons. We assume that the differences *d*_(1, *z*)*ir *_are (or transformed to be) normally distributed and the first level of our hierarchical model can be represented by

*d*_(1, *z*)*ir *_~
*MVN*(*λ*_(1, *z*)*i*_,
**Σ**)

where *λ*_(1, *z*)*i *_is the mean difference between methods 1 and *z *for subject *i*, and **Σ **is the (*M *- 1) × (*M *- 1) dimensional covariance matrix at the observation difference level. Note here that the distributions of the measurements themselves *x*_*mir *_need not be normal, only the differences *d*_(1, *z*)*ir*_.

Like before, we assume the second or subject level of the hierarchical model can be given by

*λ*_(1, *z*)*i *_~
*MVN*(*υ*_(1, *z*)_,
**Φ**)

where *υ*_(1, *z*) _is the overall mean difference between methods 1 and *z*, and **Φ **is the (*M *- 1) × (*M *- 1) dimensional subject level covariance matrix. Note that *υ*_(1, *z*) _directly gives the distribution of the bias *B*_(1, *z*)_. Again, while the selected subjects themselves may not necessarily be normal, the population from which they were selected frequently can be assumed to be normal. Prior distributions are required for *υ*_(1,2)_,..., *υ*_(1, *M*)_, **Σ **and **Φ **to complete the full parameterization, and will depend on the information available.

This multivariate hierarchical Bayesian model (*HB*_2_) can also be used in the situation when the subject's underlying values can be considered exchangeably. However, in implementing model (*HB*_2_) rather than (*HB*_1_), then some parameters of potential interest that are unavailable, such as within subject variance, $s_{within\left(m\right)}^{2}$, between subject variance, $s_{between\left(k\right)}^{2}$, the intra-class correlation coefficient, *ICC*_*k*_, and the within subject covariance of measurements between methods $\tau_{\left(y,z\right)}^{2}$. However, the variance between individual measurements on the same subject is estimable.

## Results

Two separate examples are presented and analysed. The first example which also appears in Altman and Bland is that of systolic blood pressure measurements (mm Hg) made simultaneously by two observers (J & R) using a sphygmomanometer and an automatic blood pressure measuring machine (S), each making three observations in quick succession on 85 subjects [6]. The second example which is presented by Oliver and colleagues examines step counts for 9 pre-school children (aged between 3–5 years) ambulating along a straight 29 metre line at three different speeds ("walk slowly like a snail", "walk normally", "run") measured simultaneously by an observer and from three separate pedometers placed on the left and right hip and on the back of each child [15]. The data for both examples have been reproduced in Tables A1 and A2 (see Additional file 1).

In the first example each subject's underlying value was not expected to change between repeated measurements and so we model this using both multivariate hierarchical Bayesian models (*HB*_1 _and *HB*_2_). In the second example each subject's underlying value is dependent on pace, a continually changing quantity, and so only the second multivariate hierarchical Bayesian model (*HB*_2_) could be employed.

### Prior specifications

For the purpose of this paper, we use vague prior information distributions throughout. For *HB*_1_, underlying parameters *θ*_*m*_, *i *= 1,..., *N*, were assumed to follow independent normal distributions with zero mean and low precision (0.0001), and the inverse covariance matrices (**Θ**^-1 ^and **Ω**^-1 ^respectively) followed a Wishart distribution with degrees of freedom taken to equal each matrix's rank and having diagonal elements set to 0.1, and off-diagonal elements set to 0.005 [12,16]. Similarly, for *HB*_2_, underlying parameters *υ*_(1, *z*)_, *z *= 2,..., *M*, were assumed to follow independent normal distributions with zero mean and low precision (0.0001), and the inverse covariance matrices (Σ^-1 ^and Φ^-1^) followed a Wishart distribution with degrees of freedom taken to be their rank and having diagonal elements set to 0.1 and off-diagonal elements set to 0.005. However, if informative prior information is available, then this should be specified and modelled rather than using these vague priors.

### Computation

Preliminary checks of assumptions and the Bland-Altman limit of agreement graphs were undertaken using Stata version 9.2 [17]. Numerical results from the multivariate hierarchical Bayesian models were derived from computer simulation in WinBUGS [16] (see Additional file 2). Simulations of size *N *= 50,000 were run in four parallel chains (with over-relaxation) after a burn-in period of 10,000 iterations and samples from every 10^th ^iteration thereafter was stored and utilised. Convergence in the final samples was checked using visual plots of simulation histories and the modified Gelman-Rubin statistic [18]. Reported 95% credible regions (95% CR) corresponded to the 2.5 and 97.5 percentiles of the posterior distribution of the variable of interest.

### Systolic blood pressure measurements example

**Figure 1:**
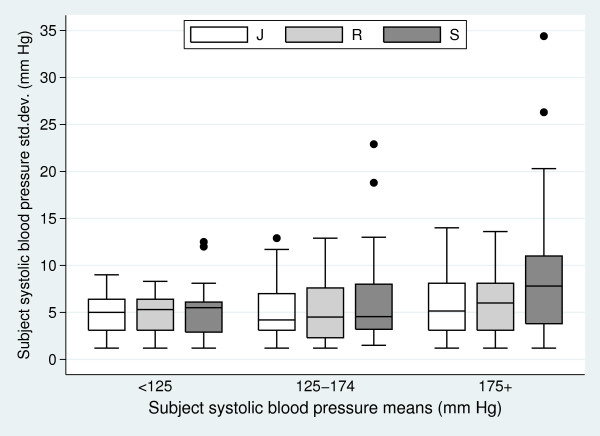
**Box-plot of the subject's systolic blood pressure measurement standard deviations by their mean systolic blood pressure measurements grouped into three categories (< 125 mm Hg, 125–174 mm Hg, and 175+ mm Hg) for the two observers (J and R) and the automated machine (S)**.

Before implementation of the multivariate hierarchical Bayesian models, a check of the assumptions was undertaken. For *HB*_1 _the subject variances should be independent of their mean for each method while for *HB*_2 _the subject paired difference variances should be independent of the subject paired difference means. Figure 1 presents this check for *HB*_1 _using box-plots of the subject standard deviations broadly grouped into three categories by their means.

**Figure 2:**
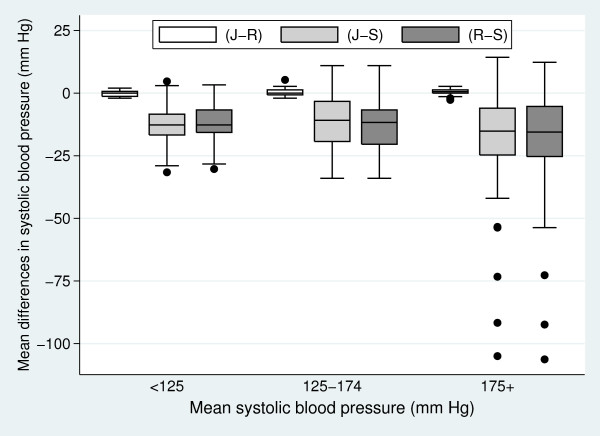
**Box-plot of subject's mean difference between systolic blood pressure measurements (mm Hg) by their mean systolic blood pressure measurements grouped into three categories (< 125 mm Hg, 125–174 mm Hg, and 175+ mm Hg) for pair-wise comparisons (J and R), (J and S), and (R and S)**.

Perusal of Figure 1 reveals that observer J and R have subject variances relatively independent from their means. This assumption of independence appears less reasonable for the automated machine (S), particularly in the higher mean grouping. Nonetheless, like Bland and Altman [6], we consider this violation to be sufficiently small as not to warrant data transformation investigations. Next we plot the difference between the subject means for each pair-wise method comparison against their average (Figure 2). The assumption of independence again appears reasonable in Figure 2. Notable also in this figure is that the median and variability of the differences between observers J and R is substantially less than those involving the automated machine S.

Table 1 presents the mean estimate and associated 95% CR of the overall population mean, *θ*_*m*_, within subject variance, $s_{within\left(m\right)}^{2}$, between subject variance, $s_{between\left(m\right)}^{2}$. and intra-class correlation coefficient, *ICC*_*m*_, for systolic blood pressure measured by two observers and the automated machine (*m *= J, R and S). These estimates were derived from *HB*_1 _using WinBUGS program Ex.1 (see Additional file 2). The estimates of $s_{within\left(m\right)}^{2}$ are similar but slightly higher than the $s_{within\left(J\right)}^{2}$ = 37.4, $s_{within\left(R\right)}^{2}$ = 38.0, and $s_{within\left(S\right)}^{2}$ = 83.1 reported by Bland and Altman for these data [6]. As previously concluded by these authors, we can see that both observers have considerably better repeatability than the machine and that the observer performance is almost identical. Additionally provided by our calculations are the $s_{between\left(m\right)}^{2}$ and *ICC*_*m *_mean estimates together with their associated 95% CR. From this we can see that the between subject variability accounts for most of the measurement variance and reliability, as measured by the *ICC*_*m*_, is high for all methods, albeit relatively higher for J and R compared to S.

**Table 1:** Posterior mean estimate and associated 95% credible region (95% CR) of the overall population mean.$s_{within\left(m\right)}^{2}$$s_{between\left(m\right)}^{2}$

	Observer J	Observer R	Machine S
	Post. mean (95% CR)	Post. mean (95% CR)	Post. mean (95% CR)
*θ*_*m*_	126.9 (120.6, 133.5)	126.9 (120.5, 133.3)	142.6 (135.9, 149.3)
$s_{within\left(m\right)}^{2}$	37.7 (30.5, 46.6)	38.3 (31.0, 47.3)	83.9 (67.9, 104.0)
$s_{between\left(m\right)}^{2}$	944.7 (697.3, 1278.0)	926.4 (683.3, 1254.0)	992.6 (727.9, 1351.0)
*ICC*_*m*_	0.96 (0.95, 0.97)	0.96 (0.94, 0.97)	0.92 (0.89, 0.95)

θm, within subject variance, $s_{within\left(m\right)}^{2}$, between subject variance, $s_{between\left(m\right)}^{2}$, and intra-class correlation coefficient, ICCm, for systolic blood pressure measured by two observers and the automated machine (m = J, R and S) from the multivariate hierarchical Bayesian method that assumes the underlying values remain static (HB1).

Estimates and associated 95% CR of bias, *B*_(*y*, *z*)_, together with estimates of the 95% limits of agreement for pair-wise comparisons of systolic blood pressure for each pair-wise comparison derived from both *HB*_1 _and *HB*_2 _are presented in Table 2.

**Table 2:** Estimates of bias, *B*_(*y*, *z*)_, and the associated 95% credible regions (95% CR) together with estimates of the 95% limits of agreement for pair-wise comparisons of systolic blood pressure measured by the two observers and the automated machine (*m *= J, R and S) from two multivariate hierarchical Bayesian models.

	J vs. R	J vs. S	R vs. S
	Mean (95% CR)	Mean (95% CR)	Mean (95% CR)
Bias, *B*_(*y*, *z*)_			
*HB*_1_	0.08 (-0.21, 0.37)	-15.6 (-19.7, -11.6)	-15.7 (-19.8, -11.7)
*HB*_2_	0.09 (-0.20, 0.37)	-15.4 (-19.5, -11.2)	-15.5 (-19.5, -11.3)
95% limits of agreement			
*HB*_1_	(-4.36, 4.56)	(-56.2, 25.1)	(-56.0, 24.5)
*HB*_2_	(-4.39, 4.57)	(-55.9, 25.0)	(-55.7, 24.6)
Within subject covariance of measurements between methods $\tau_{\left(y,z\right)}^{2}$*			
*HB*_1_	35.5 (28.5, 44.1)	16.1 (7.7, 26.6)	17.4 (8.9, 27.1)

*Note: larger within subject covariance values are desirable.

*HB*_1 _which assumes that the underlying values remain static and uses the raw data, *x*_*mir*_, and *HB*_2 _which assumes that the underlying values can continually change and uses paired difference data, *d*_(*y*, *z*)*ir*_.

It can be seen from Table 2 that there was evidence of systematic bias between the observers and the machine but not between observers. The observers read systolic blood pressure measurements on average 15.7 mmHg lower than the automated machine. In addition, Table 2 includes estimates and associated 95% CR of within subject covariance of measurements between methods $\tau_{\left(y,z\right)}^{2}$ for each pair-wise comparisons derived from *HB*_1_.

**Figure 3:**
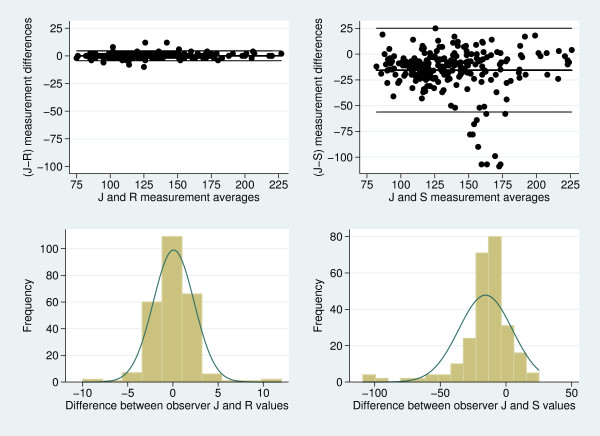
**Scatter-plots of measurement differences against measurement averages with the 95% limits of agreement superimposed (upper sub-plots) and histogram of measurement differences (lower sub-plots) for pair-wise comparisons of systolic blood pressure between the two observers (J & R), and between observer J and the automated machine S (J & S)**. The 95% limits of agreement appear in as the outer lines while the mean estimate of bias is given by the intermediate lines in the upper sub-plots.

Figure 3 depicts plots of the 95% limits of agreement and histogram of measurement differences for pair-wise comparisons of systolic blood pressure between the two observers (J & R) and between observer J and the automated machine S (J & S). The comparison between observer R and the automated machine S was very similar to the (J & S) comparison and thus not shown. For the comparison between J and R, the points on the plot are without obvious pattern and the histogram appears normal, consistent with the model's assumptions. However, when investigating the J and S comparison, there appears to be a cluster of discordant observations while the majority appear consistent with the statistical model's assumptions. Bland and Altman note that departures from normality between method differences will not have a great impact on the limits of agreement [6]. Nonetheless, investigation and verification of the data would be useful in such circumstances, as would sensitivity analyses (by removing some of the extreme data and determining their effect on the quantified level of agreement). Should the analyses be sensitive to outlying values then alternative methods of analysis need to be entertained, such as non-parametric methods or survival-agreement plots [6,9].

The limits of agreement presented in Table 2 are similar when each observer is compared to the machine. If differences within these limits of agreement are not clinically important, then we could use the two measurement methods interchangeably. In comparing observer J with the machine S, Bland and Altman calculate a bias of -15.6 and 95% limits of agreement of (-56.7, 25.4), similar to those derived from our model.

A sensitivity analysis was undertaken by removing the 8 most aberrant data that appeared in Figure 3 and repeating the analysis. These 8 outlying measurements were all recorded by machine S and were for all 3 repeated measurements for subjects 78 and 80, and 2 of the 3 repeated measures for subject 68. The subject clustered nature of the aberrant data measured from the automated machine S suggests that the device was not properly fitted or functioning for these particular subjects and behoves further investigation. For the observer J vs. machine S comparisons, the estimated bias and 95% CR was -13.1 (-15.8, -10.4), little different from that reported in Table 2. The 95% limits of agreement was estimated as (-41.1, 15.2), an interval width of 56.3 mmHg and some 31% less than that reported in Table 2 for the full data. Similarly for the observer R vs. machine S comparisons, the estimated bias and 95% CR was -13.2 (-15.8, -10.6), and 95% limits of agreement was estimated as (-40.7, 14.6), an interval width also 31% less than that reported in Table 2. While noticeable, these are not particularly large reductions in the 95% limits of agreement interval widths.

Also included in Table 2 are the results from repeating the analysis of the full systolic blood pressure data except now employing the multivariate hierarchical Bayesian model that uses paired differences (*HB*_2_). The bias estimates, their associated 95% CR, and the 95% limits of agreement are strikingly similar to those derived from *HB*_1 _due to the balance and completeness of the data.

### Step count measurements example

Step counts are believed to be dependent on the pace that pre-school children ambulate. In this study the mean (standard deviation) steps counted and recorded by the observer was 60.9 (10.7) for normal pace, 47.2 (6.9) when running, and 69.9 (7.8) at a slow walk. Because subjects underlying values are a changing quantity, we analyses these data using the non-exchangeable multivariate hierarchical Bayesian model (*HB*_2_).

A plot of the subject paired difference variances against the subject paired difference means provided no reason to refute the assumption that observations at the first level were independent (figure not shown). Implementing program Ex.2 (see Additional file 2), estimates of bias, *B*_(*y*, *z*)_, and the associated 95% CR together with estimates of the 95% limits of agreement for pair-wise comparisons of step counts measured by one observer (O) and pedometers located on the left hip (P_LH_), the right hip (P_RH_) and on the back (P_B_) were yielded and appear in Table 3.

**Table 3:** Estimates of bias, *B*_(*y*, *z*)_, and the associated 95% credible regions (95% CR) together with estimates of the 95% limits of agreement for pair-wise comparisons of step counts.

	Bias, *B*_(*y*, *z*)_		95% limits of agreement
Pair-wise comparisons (*y*, *z*)	Mean	(95% CR)	
O vs. P_LH_	-0.22	(-2.83, 2.28)	(-13.7, 13.2)
O vs. P_RH_	-0.89	(-3.48, 1.70)	(-14.6, 12.8)
O vs. P_B_	-2.66	(-5.39, 0.06)	(-17.2, 11.9)
P_LH _vs. P_RH_	-0.67	(-3.12, 1.85)	(-13.5, 12.2)
P_LH _vs. P_B_	-2.44	(-4.81, -0.05)	(-14.9, 10.1)
P_RH _vs. P_B_	-1.77	(-3.72, 0.14)	(-12.0, 8.5)

Measured by one observer (O) and pedometers located on the left hip (P_LH_), the right hip (P_RH_) and on the back (P_B_) at three different paces (normal walk, running, slow walking) on 9 pre-school children from *HB*_2 _which assumes that the underlying values can continually change and uses paired difference data, *d*_(*y*, *z*)*ir*_.

**Figure 4:**
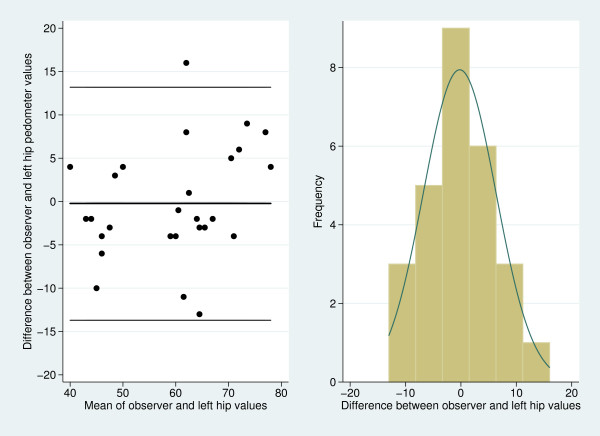
**Scatter-plots of measurement differences against measurement averages with the 95% limits of agreement superimposed and histogram of measurement differences for comparisons of step counts between the observer and the left hip placed pedometer**. The 95% limits of agreement appear as the outer lines in the left hand figure while the mean estimate of bias is given by the intermediate line.

The estimates of bias suggest that on average the pedometers undercount the observer ascertained step count, although the undercount is small for the left hip (P_LH_) and right hip (P_RH_) pedometers. Figure 4 presents the 95% limits of agreement plot and histogram of measurement differences for comparisons of step counts between the observer and the left hip placed pedometer. The points on the plot are without obvious pattern and the histogram appears normal, consistent with the model's assumptions. Plots and histograms for the other pair-wise comparison were similar and also raised no concerns about the model's assumptions (figures not shown).

The 95% limits of agreement interval widths for the observer vs. pedometer step counts were approximately 27 steps for left hip (P_LH_) and right hip (P_RH_) pedometers and 29 steps for the back (P_B_) pedometer over observed step counts that ranged on the 40 to 80 step interval. Based on this information the researcher can now decide whether differences within these limits of agreement are important for the purpose of her investigation.

## Discussion

There are many appropriate non-Bayesian methods for assessing agreement [3], however no Bayesian methods have yet been advocated or utilised. In this paper we present and employ such a method that is based on the Bland-Altman limits of agreement framework. This framework was adopted because of its simplicity, practicality, ability to detect bias and current popularity [3,10]. Moreover we concur with Bland and Altman in thinking that there is no place for methods of analysis based on hypothesis tests in assessing agreement [6]. We assert that agreement is not something that is present or absent, but something which must be quantified. Once quantified, expert judgement should be used to determine whether the estimated level of agreement is satisfactory or not for the purposes of the researcher or practitioner.

Using this limits of agreement framework, multivariate hierarchical Bayesian models provide an attractive alterative to the existing suite of analytic methods in measuring agreement using repeated measurement method comparison studies. The proposed Bayesian models are flexible, easily conceptualised and implemented (even when there are multiple measurement methods) and provide results that are intuitive and meaningful [12-14]. The ease of implementation is not limited or complicated by the balance of replicates measurement numbers made on subjects within or between methods. Moreover, the proposed models can be extended to include different parameterisations and distributional forms, any prior information available about the agreement of the methods under investigation, and include regression approaches [12-14,16]. For instance, in the second example, a regression approach could have been adopted treating pace as a covariate.

Another salient strength of the proposed hierarchical Bayesian models is that marginal distributions of the parameters of interest are yielded, thereby allowing the determination and reporting of location and scale (such as credible interval) estimates. For instance, using the exchangeable multivariate hierarchical Bayesian method (*HB*_1_), the within and between subject variances and covariance estimates and 95% credible regions were easily determined, as were intra-class coefficients. Because of the complicated distributional forms of many of these statistical parameters, 95% confidence intervals are not always readily available when using non-Bayesian statistical methods. It is of interest to note that there was a high degree of similarity between the estimates calculated and reported using Bland and Altman's methods that could be directly compared to the estimates derived from the implemented multivariate hierarchical Bayesian models with vague priors. This provides reassurance and confidence for users of either or both statistical approaches.

The properties associated with our advocated Bayesian method are not always enjoyed when using non-Bayesian software. For example, when the SAS program proposed by Carstensen and colleagues is employed for the same (J & S) comparison provided in the first example, the program fails to find a solution [11]. While Carstensen and colleagues' Stata program does find a solution, care must be taken in assigning indicator values (i.e. there is a need to order methods by their empirical variance) and not all confidence intervals for the parameters of interest are readily available. Moreover, it is unclear how the code can be generalised to the comparison of *M *> 2 methods. In the non-exchangeable measurement situation there is little in the literature guiding non-Bayesian analysts. Bland and Altman outlined one method but the specifics were lacking and no examples were provided here or elsewhere [6].

In developing the hierarchical Bayesian models, we chose to employ multivariate likelihood functions. There are many examples of multivariate hierarchical Bayesian analysis of repeated measurements outcome data already successfully employed in the medical literature [19-21]. We believe the utilisation of multivariate models is better than the successive pair-wise comparison approach presented by Bland and Altman for a number of reasons. These include the fact that there are frequently more than two methods under consideration, all information is analysed simultaneously (giving greater power, and increased statistical robustness and efficiency), marginal distributions of the parameters of interest are easily derived without asymptotic approximation, and the probabilistic approach more closely resembles to how researchers think. The modelling and implementation of the multivariate hierarchical Bayesian model with vague prior information was also straightforward. Using the freely available WinBUGS software [16] and included computer programs, computations were generally completed within minutes. If, indeed, one of the primary reasons why so few repeated measurement method comparison studies have been undertaken is due to the lack of statistical machinery readily available for the analysis of such data, then we hope our careful presentation of the statistical analysis and computer code for two examples will help circumvent this barrier for future researchers.

The proposed approach is not without its limitations. However, many of the same limitations plague the previously described limits of agreement methods. The assumption of normality may, at times, be untenable and the data require transformation or different likelihood distributions explored. The latter is perhaps hampered by the available of multivariate normal, multivariate Student t, Wishart and Dirichlet continuous multivariate distributions in WinBUGS [16]. However, as Bland and Altman note, and seen within our example, departures from normality between method differences will not usually have a great impact on the limits of agreement [6]. Nonetheless, investigation and verification of the data would be useful in such circumstances, as would sensitivity analyses, and alternative methods of analysis [6,9]. Finally, if the number of subjects and replications is few (i.e. two measurements per subject with some missing values), there is little or no prior information, and the within and between subject correlation high, then there may be considerable autocorrelation in the WinBUGS numerical simulation. Careful attention needs to be given to the simulation diagnostics, the length of the burn-in time and use of the over-relaxed form of the Markov chain Monte Carlo simulation method.

## Conclusion

Repeated measurement method comparison studies quantify the agreement between the various methods under consideration and measure the agreement each method has to itself. While both are fundamentally important measures of agreement, few studies have adopted the use of replicates to measure the latter. We present two Bayesian methods of analysis that complement those already founded in the literature [6], one which assumes that the underlying values remain static and one assuming that the underlying values can change between measurement waves. The models are easily implemented and produce readily interpretable results. We believe that these models will provide important additions to the current measurement method comparison study analytic suite and hope that this will impel researchers to conduct such studies using replicated measurements in the future.

## Competing interests

The author declares that they have no competing interests.

## Authors' contributions

PJS conceived the study, analysed and interpreted all data, and drafted the paper. The author read and approved the final manuscript.

## Pre-publication history

The pre-publication history for this paper can be accessed here:

http://www.biomedcentral.com/1471-2288/9/6/prepub

## Supplementary Material

Additional file 1 **MS-EXCEL sheet of the data used for the two examples**. This is a MS-EXCEL 97–2003 file that includes two sheets (Example 1 and Example 2) which contain the data used for the examples. The sheet entitled Example 1 gives the systolic blood pressure measurements made simultaneously by two observers (J and R) and an automatic blood pressure machine (S), each making three observations in quick succession. The sheet entitled Example 2 gives step count for 9 pre-school children (aged between 3–5 years) ambulating along a straight 29 metre line at three different speeds ("walk slowly like a snail", "walk normally", "run") measured simultaneously by an observer and from three separate pedometers placed on the left and right hip and on the back of each child. Click here for file

Additional file 2 **WinBUGS programs used for the two examples**. In a Microsoft Office Word 97–2003 document the two WinBUGS programs are presented (Program Ex.1 or Program Ex.2). Program Ex.1 was used for the exchangeable hierarchical multivariate Bayesian model (*HB*_1_) for comparison of systolic blood pressure measurements made by two observers and an automated machine. Program Ex.2 was used for the non-exchangeable hierarchical multivariate Bayesian model (*HB*_2_) for comparison of step counts made by an observer and pedometers located in three sites (right hip, left hip and back). Click here for file
