# Supplementary material for: A multivariate hierarchical Bayesian approach to measuring agreement in repeated measurement method comparison studies
Source: BMC Med Res Methodol. 2009 Jan 22;9:6. doi: 10.1186/1471-2288-9-6 (PMC2645135; doi:10.1186/1471-2288-9-6)
Supplement: Additional file 2 — WinBUGS programs used for the two examples. In a Microsoft Office Word 97–2003 document the two WinBUGS programs are presented (Program Ex.1 or Program Ex.2). Program Ex.1 was used for the exchangeable hierarchical multivariate Bayesian model (HB1) for comparison of systolic blood pressure measurements made by two observers and an automated machine. Program Ex.2 was used for the non-exchangeable hierarchical multivariate Bayesian model (HB2) for comparison of step counts made by an observer and pedometers located in three sites (right hip, left hip and back). [file 1471-2288-9-6-S2.doc]

**Program Ex.1.** *Exchangeable hierarchical multivariate Bayesian model $\left(HB_{1}\right)$ for comparison of systolic blood pressure measurements made by two observers and an automated machine*.

MODEL

model{ for (i in 1:Subjects){

for (j in 1:Replications){

x[i,j,1:3] ~ dmnorm(mu[i,1:3],P[,])}

mu[i,1:3] ~ dmnorm(theta[1:3],T[,])}

for (k in 1:3){

theta[k] ~ dnorm(0,0.0001)

ICC[k] <- V[k,k]/(V[k,k]+W[k,k])} # Intra-class correlation

P[1:3,1:3] ~ dwish(U[1:3,1:3],3)

T[1:3,1:3] ~ dwish(R[1:3,1:3],3)

W[1:3,1:3] <- inverse(P[1:3,1:3])

V[1:3,1:3] <- inverse(T[1:3,1:3])

p.mu[1:3] ~ dmnorm(theta[1:3],T[,]) # Predicted means

p.x[1:3] ~ dmnorm(p.mu[1:3],P[,]) # Predicted measurements

for (y in 1:2){

for (z in (y+1):3){

bias[y,z] <- theta[y]-theta[z] # Bias estimation

diff[y,z] <- p.x[y]-p.x[z]}}} # Limits of agreement

INITIALS

list(theta=c(127,143,143),

P=structure(.Data=c(1,0,0,0,1,0,0,0,1),.Dim=c(3,3)),

T=structure(.Data=c(1,0,0,0,1,0,0,0,1),.Dim=c(3,3)))

DATA

list(Subjects=85, Replications=3,

x=structure(.Data=c(100,98,122,106,98,...(data omitted)...,128),.Dim=c(85,3,3)),

U=structure(.Data=c(0.100,0.005,0.005,

0.005,0.100,0.005,

0.005,0.005,0.100), .Dim=c(3,3)),

R=structure(.Data=c(0.100,0.005,0.005,

0.005,0.100,0.005,

0.005,0.005,0.1), .Dim=c(3,3)))

**Program Ex.2.** *Non-exchangeable hierarchical multivariate Bayesian model $\left(HB_{2}\right)$ for comparison of step counts made by an observer and pedometers located in three sites (right hip, left hip and back).*

MODEL

model{ for (i in 1:Subjects){

for (j in 1:Replications){

d[i,j,1:3] ~ dmnorm(mu[i,1:3],P[,])}

mu[i,1:3] ~ dmnorm(theta[1:3],T[,])}

for (k in 1:3){

theta[k] ~ dnorm(0,0.0001)}

P[1:3,1:3] ~ dwish(U[1:3,1:3],3)

T[1:3,1:3] ~ dwish(R[1:3,1:3],3)

W[1:3,1:3] <- inverse(P[1:3, 1:3])

V[1:3,1:3] <- inverse(T[1:3, 1:3])

# Bias estimates

bias[1,2] <- theta[1]

bias[1,3] <- theta[2]

bias[1,4] <- theta[3]

bias[2,3] <- theta[2]-theta[1]

bias[2,4] <- theta[3]-theta[1]

bias[3,4] <- theta[3]-theta[2]

# Predictive distribution for individual measurement pair differences

p.mu[1:3] ~ dmnorm(theta[1:3],T[,])

p.x[1:3] ~ dmnorm(p.mu[1:3],P[,])

p.x[4] <- p.x[2]-p.x[1]

p.x[5] <- p.x[3]-p.x[1]

p.x[6] <- p.x[3]-p.x[2]}

INITIALS

list(theta=c(-0.22,-0.96,-2.70), P=structure(.Data=c(1,0,0,0,1,0,0,0,1),.Dim=c(3,3)),

T=structure(.Data=c(1,0,0,0,1,0,0,0,1),.Dim=c(3,3)))

DATA

list(Subjects=9, Replications=3,

d=structure(.Data=c(-10,-9,-16,1,-6,-7,-2,3,...(data omitted)...,10),.Dim=c(9,3,6)),

U=structure(.Data=c(0.100,0.005,0.005,

0.005,0.100,0.005,

0.005,0.005,0.100), .Dim=c(3,3)),

R=structure(.Data=c(0.100,0.005,0.005,

0.005,0.100,0.005,

0.005,0.005,0.1), .Dim=c(3,3)))
